# Real life pharmaceutical treatment patterns for adult patients with focal epilepsy in Germany: a longitudinal and cross-sectional analysis of recently approved anti-epileptic drugs

**DOI:** 10.3205/000250

**Published:** 2017-06-12

**Authors:** Antje Groth, Thomas Wilke, Simon Borghs, Patrick Gille, Lars Joeres

**Affiliations:** 1 Ingress Health GmbH, Wismar, Germany; 2 IPAM e.V., University of Wismar, Germany; 3 UCB Pharma ltd, Slough, UK; 4 UCB Pharma GmbH, Monheim, Germany

**Keywords:** focal epilepsy, antiepileptic drugs, drug therapy

## Abstract

**Objectives**: The aim of this study was to investigate the antiepileptic drug (AED) treatment of adults suffering from focal epilepsies (FE) in Germany. Of special interest was the number and percentage of the patients 16 years and older receiving no treatment with an AED, treatment with one AED (monotherapy), treatment with more than one AED, and treatment with a novel AED. The definition for “novel” was newly approved at the time of market entry since 2006 (last 10 years): eslicarbazepine (ESL), lacosamide (LCM), perampanel (PER), and retigabine (RTG).

**Methods:** The analysis was based on a claims data set covering the years 2007 to 2014, provided by AOK PLUS, a German statutory health insurance. Two patient samples were defined: (1) prevalent patients suffering from FE (at least one in- or outpatient diagnosis of FE and at least one prescription of an AED), and (2) incident FE patients (first in- or outpatient diagnosis of FE without any previous diagnoses/AED prescriptions in the preceding 6 months). Patient observation started at date of first observed inpatient or outpatient focal epilepsy diagnosis within the analyzed period.

Each patient was classified as a “no AED therapy”, “AED monotherapy” or “more than one AED therapy”. Patients were analyzed by number and type of concomitantly prescribed AEDs in yearly tranches (no, one, two, three, four, more than four AEDs; novel versus non-novel AEDs).

**Results:** A total of 34,422 patients diagnosed with FE aged 16 year or older (mean age 59.6 years, 48.7% female) were identified. The mean follow-up period was 1,891 days (5.2 years) since first confirmed diagnosis.

The percentage of prevalent patients diagnosed with FE who received one AED (monotherapy) was stable overall and ranged between a minimum of 66.2% (2007) and a maximum of 68.9% (2010). The percentage of patients who received two AEDs ranged from 23.6% (2012) to 25.8% (2007). The remaining patients received therapies with three (6.0% in 2010 to 6.7% in 2007), four (1.0% in 2010 to 1.2% in 2009) or more than four AEDs (0.1% in 2014 to 0.3% in 2013). Between 8.1%–16.6% (2007; 2014) of the patients received no AED therapy in the observed period.

In the first year after the diagnosis of FE (incident patients), 9.7% of patients didn’t receive any AED therapy. Of those treated with at least one AED, 80.0% received one AED (monotherapy) only, 17.0% received therapy with two AEDs, 2.6% with three AEDs, 0.3% with four AEDs, and 0.1% with >4 AEDs during the respective observation time window and remained stable throughout the four-year follow-up period.

Of prevalent patients with a diagnosis of FE, 1,889 (5.5%) received at least one prescription of a novel AED during the observation period; 98.6% of these patients received the novel AED in combination with at least one other AED. Of those patients, 269 (14.2%) received >1 novel AED. The analysis of the patients receiving novel AEDs by the time from the first confirmed diagnosis of FE until the prescription of a novel AED resulted in a mean duration of 4.0 years (SD 2.0) for ESL, 3.6 years (SD 2.2) for LCM, 5.7 years (SD 1.2) for PER, and 4.6 years (SD 0.8 years) for RTG. The mean number of AEDs prescribed before the novel AEDs were 3.2 for ESL, 2.4 for LCM, 5.0 for PER and 5.2 for RTG.

**Conclusions:** Most patients aged 16 years or older, suffering from focal seizures, received AED monotherapy. Novel AEDs were prescribed in a small proportion of patients (<6%) and relatively late in the treatment course. These results are consistent with the recommendations of the German Society for Epileptology (Deutsche Gesellschaft für Epileptologie, DGfE) which suggests a number of monotherapy options – these options do not include the novel AEDs described in this study.

## Zusammenfassung

**Ziel:** Das Ziel der vorliegenden Studie war die Beschreibung der medikamentösen Therapie von erwachsenen Patienten mit fokaler Epilepsie (FE) in Deutschland. Hierbei wurden insbesondere die Patienten analysiert, die 16 Jahre oder älter sind und keine antiepileptische Medikation, eine Monotherapie, eine Therapie mit mehr als einem Wirkstoff und/oder einem neuartigen Antiepileptikum erhielten. Für die Definition von „neuartig“ wurde die Neuzulassung mit Zeitpunkt des Markteintrittes nach 2006 (in den letzten 10 Jahren) herangezogen: Eslicarbazepin (ESL), Lacosamid (LCM), Perampanel (PER) und Retigabin (RTG).

**Methoden:** Basis für die vorliegende Studie waren anonymisierte Routinedaten aus den Jahren 2007 bis 2014 der AOK PLUS, einer deutschen gesetzlichen Krankenversicherung. Es wurden zwei Patientengruppen analysiert: (1) prävalente Patienten mit FE (mindestens eine ambulante oder stationäre Diagnose FE und mindestens eine Verordnung eines Antiepileptikums) und (2) inzidente Patienten mit FE (6 Monate vor der Erstdiagnose keine andere Epilepsiediagnose und keine Verordnung eines Antiepileptikums). Die jeweiligen Patienten wurden ab der ersten ambulanten oder stationären Diagnose mit FE innerhalb des Analysezeitraums beobachtet.

Jeder Patient wurde in Hinblick auf folgende Behandlungsmuster analysiert: „keine antiepileptische Medikation“, „Monotherapie“ oder „Therapie mit mehr als einem antiepileptischen Wirkstoff“.

In der letztgenannten Gruppe wurden die Patienten nach der Anzahl und Art der verordneten antiepileptischen Wirkstoffe innerhalb eines Jahres weiter differenziert (zwei, drei, vier, mehr als vier Antiepileptika).

**Ergebnis:** Insgesamt wurden 34.422 Patienten mit FE (Durchschnittsalter 59,6 Jahre; 48,7% weiblich) mit einer durchschnittlichen Beobachtungsdauer von 1.891 Tagen (5,2 Jahre) identifiziert.

Der Anteil prävalenter Patienten, die eine Monotherapie erhielten, war über den Beobachtungszeitraum konstant mit einem Minimum von 66,2% (2007) und einem Maximum von 68,9% (2010). Der Anteil der Patienten mit zwei verordneten antiepileptischen Wirkstoffen in einem Jahr lag zwischen 23,6% (2012) und 25,8% (2007). Die verbleibenden Patienten erhielten drei (von 6,0% in 2010 bis 6,7% in 2007), vier (von 1,0% in 2010 bis 1,2% in 2009) oder mehr als vier verschiedene Antiepileptika (zwischen 0,1% in 2014 und 0,3% in 2013). Zwischen 8,1% (2007) und 16,6% (2014) der Patienten erhielten keine antiepileptische Medikation im Beobachtungszeitraum.

Im ersten Jahr nach Erstdiagnose erhielten 9,7% der inzidenten Patienten keine antiepileptische Medikation. Patienten, die mindestens eine Verordnung eines Antiepileptikums erhielten, wurden zu 80,0% als Monotherapie-Patienten identifiziert, 17,0% erhielten eine Therapie mit zwei antiepileptischen Wirkstoffen, 2,6% mit drei Wirkstoffen, 0,3% mit vier Wirkstoffen und 0,1% der Patienten erhielten mehr als vier unterschiedliche Antiepileptika. Die Verteilung von Monotherapie und Therapie mit zwei oder mehr Wirkstoffen war über die gesamte vierjährige Beobachtungszeit konstant.

Insgesamt erhielten 1.889 prävalenten Patienten mit FE (5,5%) mindestens eine Verordnung eines neuartigen Antiepileptikums (98,6% in Kombination mit mindestens einem anderen Antiepileptikum). Von diesen Patienten erhielten 269 Patienten (14,2%) mindestens zwei neuartige Antiepileptika. Die durchschnittliche Dauer zwischen Epilepsie-Erstdiagnose und Erstverordnung eines neuartigen antiepileptischen Wirkstoffs betrug bei ESL 4,0 Jahre (SD 2,0), bei LCM 3,6 Jahre (SD 2,2), bei PER 5,7 Jahre (SD 1,2) und RTG 4,6 Jahre (SD 0,8). Vor der Erstverordnung eines neuartigen Antiepileptikums wurden durchschnittlich 3,2 (ESL), 2,4 (LCM), 5,0 (PER) und 5,2 (RTG) andere antiepileptische Wirkstoffe verordnet.

**Schlussfolgerung: **Die meisten Patienten mit FE im Alter von mindestens 16 Jahren erhalten eine antiepileptische Monotherapie. Neuartige Antiepileptika werden nur bei einem geringen Teil der Patienten (<6%) und relativ spät im Behandlungsverlauf verordnet. Die Ergebnisse entsprechen der deutschen Leitlinie zur Behandlung von Epilepsien (Deutsche Gesellschaft für Epileptologie, DGfE). Diese empfiehlt verschiedene Monotherapieoptionen – neuere Antiepileptika, wie in dieser Studie analysiert, sind nicht darunter.

## Background

Epilepsy is one of the most frequent neurological diseases, with an estimated prevalence of 0.5–1.0% of the general population [1], [2]. Based on that estimation, 400–800,000 people suffer from epilepsy in Germany [2], [3]. Epilepsy is characterized by recurrent and unprovoked seizures caused by abnormal transmission of electrical signals and neuronal activity in the brain [1], [4]. The major categories of seizure type were classified by the International League Against Epilepsy (ILAE) as focal (partial) seizures and generalized seizures [5]. Studies have found 33–65% of those with epilepsy to have focal seizures or localization-related epilepsies [6]. Generally, about 50–70% of patients achieve seizure remission on an initial or secondary antiepileptic drug (AED) as monotherapy. Seizure remission in another 15–25% of patients may be obtained with AED combination therapy, with the remaining 5–35% of patients failing to achieve satisfactory seizure remission [1], [7], [8]. Combination therapy with a variety of AEDs is considered standard of care for these patients. This may also include combination therapy with novel AEDs (eslicarbazepine [ESL], lacosamide [LCM], perampanel [PER] and retigabine [RTG]) which were introduced since 2006 (in the last decade) and which have shown to be associated with improved seizure control in patients with focal epilepsy (FE) [9], [10], [11], [12], [13], [14], [15], [16], [17], [18],[19], [20].

No data are currently available on the percentage of FE patients being prescribed a combination therapy and/or a combination therapy that includes novel AEDs in German clinical practice. In a recent observational study, Steinhoff et al. analyzed treatment of German epilepsy patients having received an add-on treatment with PER. Among these, about 16% received an AED monotherapy when PER treatment was started, 40% received a combination of two AEDs, 22% of three AEDs, and 20% of more than four AEDs [21]. No known real-world study so far has addressed the treatment of FE patients based on the AEDs most recently introduced to the German market.

Consequently, the aim of this study was to describe the AED treatment of patients with FE in Germany, considering all AEDs available on the German market and approved for treatment of FE and compare these results qualitatively to treatment guidelines. Because there are different requirements for treatment and management of epilepsy in children, in this study only patients aged 16 years and older at index date were analyzed. The European Medicines Agency (EMA) has set the upper age limit for pediatric patients at 16–18 years [22]. For the purpose of this study, 16 years has been set as the lower age limit of adulthood [23], [24], [25], [26]. 

The decision to examine two patient samples (prevalent and incident) as well as to apply two different types of AED analysis (by number of AEDs and by novel AED versus non-novel AED) was undertaken so as to assess the potential differences in AED treatment approach. Factors that could potentially influence AED selection and treatment include (1) the rising availability of different AEDs and presumed changing prescription behavior over the last ten years, (2) the individual treatment journeys newly diagnosed patients (incident FE patients) experience, and (3) the treatment history of patients before they receive a novel AED.

## Methods

### Dataset and sample definition

The claims data set was provided by AOK PLUS, a German statutory health insurance fund which currently insures about 3 million people by statute in Germany. The dataset contained information on the socio-demographic characteristics of the patients and claims related to their pharmaceutical, outpatient and inpatient treatment for the years 2007–2014 (anonymized data).

Two patient samples were observed: (1) adult prevalent FE patients and (2) adult incident FE patients. A patient was included in the prevalent FE sample if he or she received at least one outpatient or inpatient diagnosis of FE (ICD10-Code G40.0/G40.1/G40.2) in the period 01/01/2007–31/12/2013. Only patients at least 16 years of age at index date and continuously insured between 01/01/2007 and 31/12/2014 were included (death was no exclusion criterion). In addition, at least one prescription of an AED (Table 1 (Tab. 1)) during 01/01/2007–31/12/2014 was required.

A patient was determined as being FE-incident if he or she received the first (‘incident’) outpatient or inpatient diagnosis of FE in the period between 01/07/2007 and 31/12/2011 (ICD10-Code G40.0/G40.1/G40.2); ‘first’ being determined by the absence of any FE diagnosis and absence of any AED prescription in the six months preceding the date of the incident diagnosis. Again, at least one prescription of an AED (Table 1 (Tab. 1)) during 01/01/2007–31/12/2014 was required. The incident FE sample represents a subgroup of the prevalent FE patients.

### Treatment of adult prevalent FE patients

For adult prevalent FE patients, AED treatment was described separately for each of the calendar years, 2007–2014. A patient was only included in the treatment analysis of a specific year if he or she received at least one diagnosis of FE in the respective or one of the earlier years. Patients who received either their FE diagnosis later than in the observed year or who already died in an earlier year were excluded. Consequently, the number of observed patients varied over time. In each respective year: (1) the number of different prescribed AEDs, (2) name of prescribed AEDs, and (3) prescribed dosage per drug, based on the “daily defined dose” (DDD) for each drug/package size as defined by the World Health Organization (WHO) and modified for Germany by the “Wissenschaftliches Institut der AOK” (WIdO) were analyzed [27], [28]. 

Each patient was classified either as a “no AED therapy patient”, “AED monotherapy patient” or “patient who received more than one AED”; in the latter group patients were differentiated by number of different prescribed AEDs during the year (two, three, four, more than four):

- “No AED therapy”: no prescription of any AED in the observed calendar year;
- “AED monotherapy”: at least one prescription of one specific AED throughout the whole calendar year;
- “More than one AED”: at least one prescription of at least two different AEDs in the observed calendar year (the specific number of different AEDs defines the respective therapy group).

For the last observation year (2014) an in-depth analysis of all patients with a combination therapy of at least two AEDs was done. For every patient, it was determined how many different AEDs they had used before they achieved the combination treatment in 2014. Within that classification, it was assessed which AEDs these patients received in 2014.

### Treatment of incident adult patients suffering from FE

For adult incident patients, AED treatment over time since the incident diagnosis of FE (index date) was described. For each patient, the treatment in the first, second, third, and fourth follow-up year since index date was analyzed. For these years, the treatment groups as defined above (“no AED therapy”, “AED monotherapy”, “more than one AED”) were assessed. In a sensitivity analysis, the DDD-based overlap of prescribed AEDs in patients who received more than one AED was analyzed. In this specific analysis, adjunctive therapy was assumed if the date of prescription of another AED occurred during the imputed availability of a previous prescribed AED. The availability was calculated as date of prescription plus DDD of the prescribed package.

### Treatment with novel AEDs

AEDs that were introduced into the German market from 2006 to 2014 were characterized as “novel AEDs”. Four agents fulfilled this criterion (ESL, LCM, PER, RTG). Among these, PER and RTG were only recently introduced to the market (2012 and 2011) and were subsequently taken from the market (PER in 2014 and RTG in 2012) due to pricing negotiations as a consequence of the recently introduced AMNOG (Arzneimittelmarkt-Neuordnungsgesetz) law. The “AMNOG”, (Act on the Reform of the Market for Medicinal Products) is a German law, which came into effect in 2011. It regulates pricing determinations for newly authorized pharmaceutical products and their reimbursement, based on an assessment of clinical additional benefit against a defined appropriate comparator.

For prevalent patients with a diagnosis of FE who received at least one prescription of a novel AED, the time from the first observed FE diagnosis until the first observed prescription of a novel AED was assessed. Furthermore, both the number and type of previously prescribed AEDs (before date of first prescription of novel AED), as well as any additional AEDs prescribed on or after the date of the first novel AED prescription in the respective year, were described. Because of very low sample sizes (<10 patients in agent-specific patient groups), these analyses were not repeated for adult incident FE patients. 

### Regulatory aspects

As the study addressed a retrospective, anonymized dataset, no ethical review and no informed consent of patients were warranted. However, the study protocol was reviewed by a scientific steering committee to which all the authors as well as the data provider, AOK PLUS, belonged.

## Results

### Patient samples and descriptive characteristics

**Figure 1:**
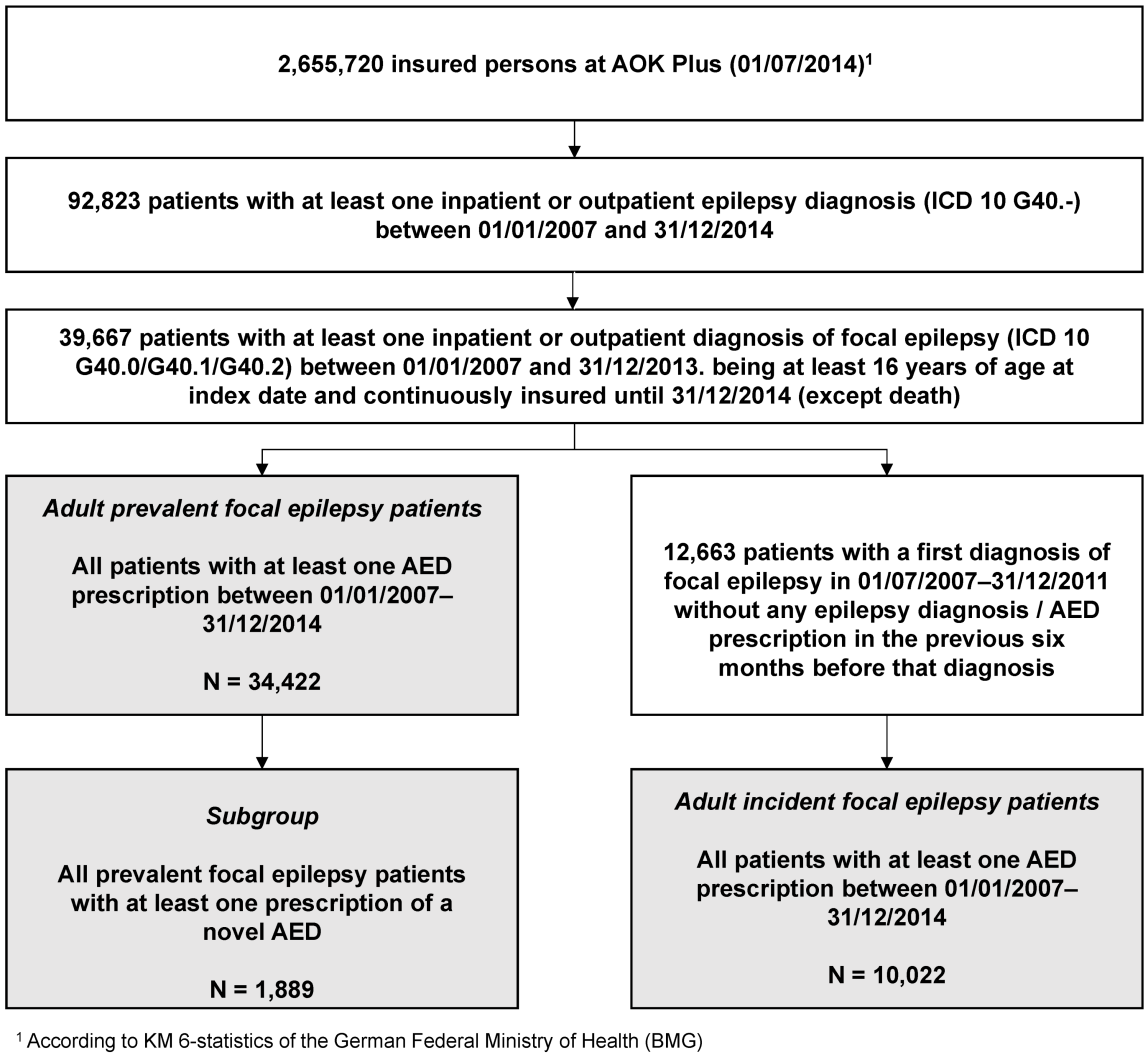
Definition of the study population Fig. 1 shows a flow chart of the patients (16 years and older) identified and included in the analyses. Please note that the main samples analyzed were prevalent FE patients and incident FE patients (one diagnosis of FE and, additionally, at least one AED prescription throughout the whole observational period).

Applying the inclusion criteria, 39,667 patients (aged 16 years and older at index date) with at least one outpatient or inpatient diagnosis of FE between 01/01/2007 and 31/12/2013 were identified (Figure 1 (Fig. 1)). Out of those patients, 86.8% (34,422) received at least one AED prescription at any time during the entire observation period; representing the final adult prevalent FE sample. The mean follow-up period for these patients was 1,891 days (5.2 years). Prescription of a novel AED occurred in 5.5% (1,889) of patients, for whom the mean follow-up time was 2,334 days (6.39 years) (Table 2 (Tab. 2)).

Among all adult patients with at least one diagnosis of FE, 12,663 patients received their first epilepsy diagnosis without any prior epilepsy diagnosis or AED prescription in the preceding 6 months. Of those, 10,022 patients (79.1%) received at least one AED prescription throughout the observation period; defined as the incidence sample. The mean follow-up period for this sample was 1,499 days (4.1 years) (Figure 1 (Fig. 1), Table 2 (Tab. 2)).

Mean age in the adult prevalent FE sample was 59.6 years (SD 19.9), 51.7 years in the novel AED-subgroup (SD 17.8), and 63.3 years (SD 20.1) in the incident sample (Table 1 (Tab. 1)). The proportion of patients aged over 70 years was exceptionally high in the sample of incident patients: 47.4% versus 37.2% in the prevalent sample. Gender was distributed similarly between the groups: 48.7% females in the sample of prevalent patients, 52.5% females in the novel AED-subgroup, and 49.1% females in the sample of incident patients (Table 2 (Tab. 2)).

### Drug treatment of adult prevalent FE patients

**Figure 2:**
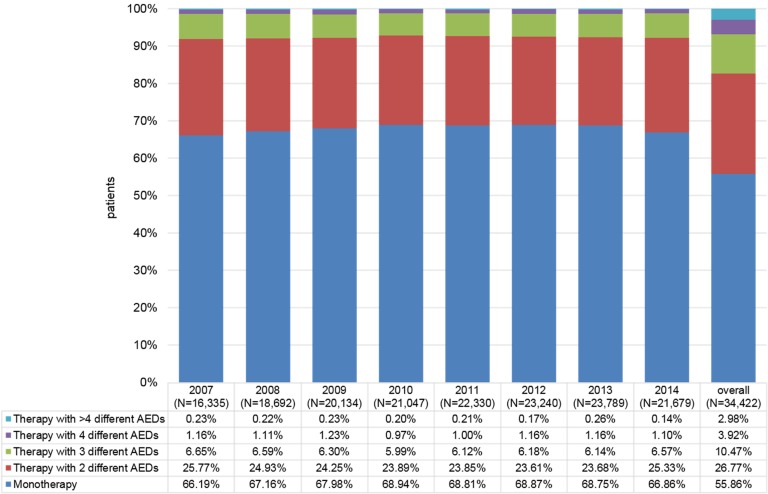
Treatment patterns of adult prevalent FE patients Fig. 2 shows the percentage of prevalent FE patients who received one AED (monotherapy) or a therapy with >1 AED. Please note that this is based on an observation of AED prescriptions for each respective calendar year. So, monotherapy required that only one AED was prescribed throughout the whole observed calendar year.

Based on the applied inclusion criteria, confirmed FE was assumed to exist only if at least one diagnosis of FE and at least one prescription of an AED throughout the whole observation period were documented. Nevertheless, in the calendar year-based analysis, the percentage of diagnosed patients who did not receive any AED therapy ranged between 8.1% in 2007 to 15.4% in 2014. Considering the entire follow-up period (2007 to 2014), 55.9% of prevalent FE patients received a therapy with only one AED throughout the observation period (“AED monotherapy”) only, whereas 26.8% received an AED therapy with two, 10.5% with three, 3.9% with four, and 3.0% with more than four different AEDs (Figure 2 (Fig. 2), Table 3 (Tab. 3)). 

Considering each calendar year, the percentage of patients who received an AED as monotherapy was relatively stable and ranged between 66.2% (2007) and 68.9% (2010). For AED (monotherapy) patients, based on the DDD of the respective agents, 174–225 days of drug supply were prescribed per observed year. The percentage of patients who received a therapy with two different AEDs ranged from 23.6% (2012) to 25.8% (2007). For these patients, based on the DDD of the respective agents, 419–515 days of DDD drug supply were prescribed per patient in the observed years (sum of all DDDs of all available AEDs per patient per calendar year). The remaining patients received therapies with three different AEDs (6.0% in 2010 to 6.7% in 2007), four different AEDs (1.0% in 2010 to 1.2% in 2009) or more than four different AEDs (0.1% in 2014 to 0.3% in 2013) (Table 3 (Tab. 3)/Figure 2 (Fig. 2)).

The most frequently prescribed AED was carbamazepine (CBZ) which was prescribed in 40.4% of patients in 2007 (that percentage decreased to 21.4% in 2014), secondly valproate (VPA; 31.5% of the patients in 2007 decreasing to 25.6% in 2014), thirdly levetiracetam (LEV; 14.1% of the patients in 2007 increasing to 35.5% in 2014). LEV became the most often prescribed AED in 2014 (Table 4 (Tab. 4)). 64.1% of all CBZ-treated patients, 52.1% of the VPA-treated patients and 48.3% of all patients with LEV-treatment received the respective agent as monotherapy in the calendar year-based observation. In contrast, felbamate (FBM), LCM, mesuximide (MSM), PER, RTG, and vigabatrin (VGB) were almost exclusively prescribed adjunctive to other AEDs (proportion of monotherapy patients <2%) in any calendar year. The percentage of patients who received one of the four novel AEDs in the last observed year 2014 were 0.9% for ESL, 4.6% for LCM, 0.1% for PER (commercialization of PER was discontinued in 2013 in Germany), and 0% for RTG (commercialization of RTG was discontinued in 2012 in Germany).

A more detailed analysis of patients with prescriptions of at least two different AEDs in the last observation year (2014; N=7,185 patients) revealed that more than 50% of these patients have never discontinued an agent since start of observation; their current combination therapy is still the first observed therapy or includes all previously prescribed AEDs. On the other hand, 525 patients (7.3%) discontinued at least three different AEDs before their therapy line in 2014 (Table 5 (Tab. 5)).

### Treatment of adult incident FE patients

Considering the whole follow-up period and our sample of 10,022 adult incident FE patients who received at least one AED prescription throughout the whole follow-up period, 66.0% of these patients received an AED monotherapy exclusively, whereas 23.9% of the patients received a therapy which included two AEDs and 10.1% three or more AEDs (three AEDs: 7.0%; four AEDs: 2.0%; >4 AEDs: 1.1%), based on the observed follow-up years since the incident FE diagnosis. The number of observed patients decreased over time due to end of individual observation (end of data availability or death of the patients).

In the first year after the incident FE diagnosis, 9.7% of patients received no AED therapy. Considering only those that received at least one AED, 80.0% received a monotherapy (with a mean of 170 prescribed days of supply, based on the DDDs of the respective drugs), 17.0% received a therapy with two different AEDs (291 prescribed days of supply), 2.6% received a therapy with three different AEDs (459 prescribed days of supply), 0.3% a therapy with four different AEDs (527 prescribed days of supply), and 0.1% a therapy with >4 different AEDs (558 prescribed days of supply). Throughout the four-year follow-up period, the percentage of patients who received either monotherapy, or a therapy with at least two different AEDs, was stable. However, the mean prescribed AED dosage (reported in prescribed DDDs per year) increased, especially in the first three years (monotherapy: 170 days in first year, 192 days in second year, 199 days in third year, 194 days in fourth year; therapy with two agents: 291 days in first year, 415 days in second year, 439 days in third year, 452 days in fourth year; numbers for therapies based on three, four or >4 AEDs reported in Table 6 (Tab. 6)).

In a sensitivity analysis, the treatment of epilepsy-incident patients in their fourth follow-up year was observed with another method. In the main analysis, the proportion of patients receiving: AED monotherapy; a therapy with 2 AEDs; and 3 AEDs was 80.6% (4,345 patients); 16.4% (882 patients); and 2.5% (132 patients) respectively. In an analysis to ascertain whether the prescribed drugs for the second and third groups had any overlap in the DDD supply, which would be an indication for a true concomitant AED therapy, was undertaken. Overall, 88 patients (1.7%) who were categorized as patients with two different AEDs (86 patients) or three different agents (2 patients) without any overlap in drug supply were identified. If these were added to the monotherapy group, assuming that no overlap indicates the drugs were prescribed one after each other, the percentage of patients receiving monotherapy would increase from 80.6% to almost 82.2%. The application of this method to verify the accuracy of the calculations for the combination therapy with two and three AEDs, would result in a decrease to 15.6% from 16.4% for two AEDs and 1.9% instead of 2.4% for three AEDs.

### Treatment of adult prevalent FE patients with novel AEDs

From all adult prevalent FE patients, 1,889 patients were identified (5.5%) receiving at least one prescription of a novel AED; 269 patients received >1 novel AED. 362 patients were treated with ESL, 1,645 patients with LCM, 162 patients with PER, and 59 patients with RTG. 

Considering all patients who received a novel AED, 98.6% received this therapy as an adjunct to at least one other AED in any given year. The most frequently prescribed AEDs in association with novel AEDs were LEV, VPA and LTG (Table 7 (Tab. 7)).

**Figure 3:**
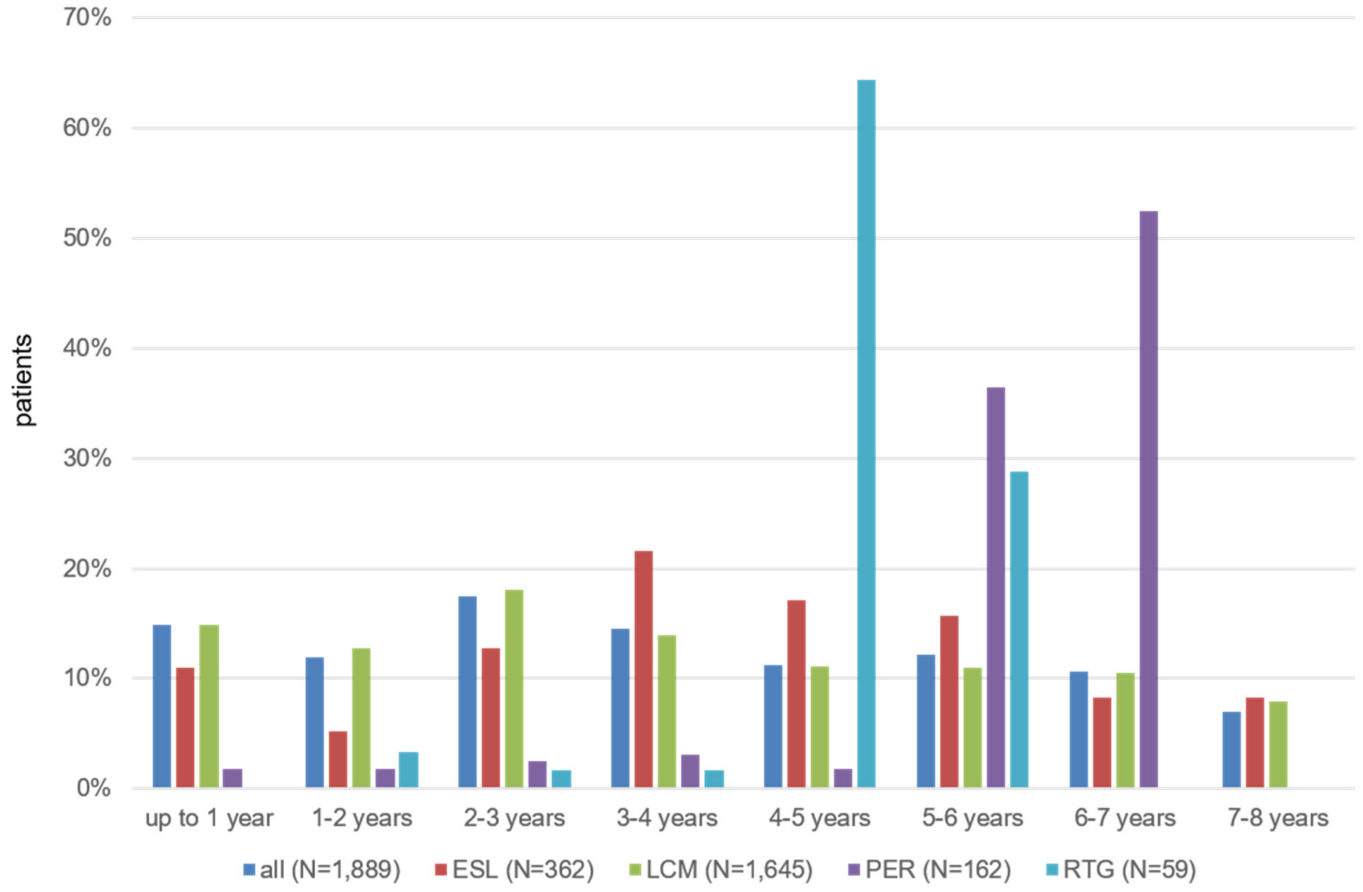
Time from first FE diagnosis to first prescription of a novel AED (based on prevalent FE patients) Fig. 3 presents the proportion of patients by time from the date of the first observed diagnosis of FE in prevalent epilepsy patients until date of first prescription of a novel AED. Only patients with at least one prescription of a novel AED were included in this analysis.

Mean duration from the date of the first observed FE diagnosis to the date of the first prescription of a novel AED was 3.59 years (SD 2.19). This time ranged from 3.58 years (SD 2.22) for LCM up to 5.69 years (SD 1.22) for PER. About one third of the patients received a novel AED only 5 years after first FE diagnosis (Figure 3 (Fig. 3), Table 7 (Tab. 7)). 

**Figure 4:**
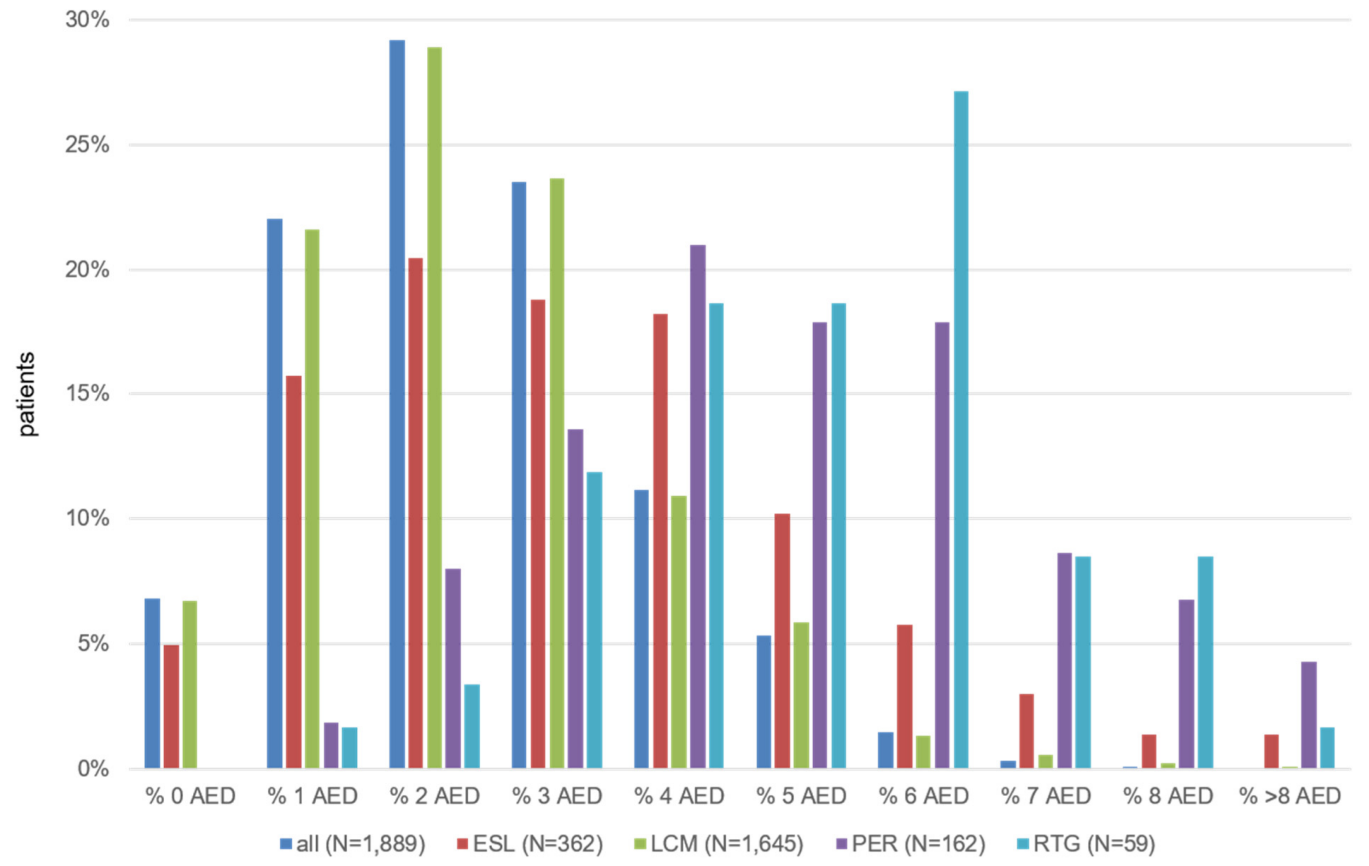
Observed number of different AEDs used before the first novel AED was prescribed (based on prevalent FE patients) Fig. 4 presents the proportion of patients by number of different AEDs used before the first novel AED was prescribed. Only patients with at least one prescription of a novel AED were included in this analysis.

Before the first novel AED was prescribed, a mean number of previously prescribed AEDs of 2.35 (SD 1.38) was identified. The drug-specific numbers were 2.39 (LCM), 3.18 (ESL), 4.96 (PER) and 5.22 (RTG). The most frequently prescribed AED prior to initiation of a novel AED therapy was LEV, followed by VPA and LTG (Figure 4 (Fig. 4), Table 7 (Tab. 7)).

## Discussion

### Results and comparison to previous literature

This study describes the real-world pharmaceutical treatment of adult patients with a diagnosis of FE in Germany, especially the use of novel AEDs (LCM, ESL, PER, RTG), and provides an interesting insight into the extent to which the German treatment guidelines are followed in clinical practice.

The results show that the majority of adult patients with FE (approximately 70%) received monotherapy during the eight years’ follow-up. Previous studies have shown that the majority of epilepsy patients achieved seizure-freedom on AED monotherapy (first- or second-line) [1], [8], [29]. In the present analysis (calendar year-based observation) about 30% of adult patients with FE received a therapy with at least two different AEDs. That percentage remained relatively stable in both the calendar year-based observation of prevalent epilepsy patients and the analysis of four years of follow-up since date of first epilepsy diagnosis for incident epilepsy patients. The percentage of patients reaching seizure-freedom could not be analyzed as data on treatment outcome were not included in the claims database. According to previous literature, it can be assumed that a considerable percentage of these patients remain uncontrolled; previous studies stated a percentage of about 30% of all epilepsy patients [29], [30], especially patients with FE, are more likely to remain uncontrolled [29], [31].

A recent German study reported that between 32% and 69% of epilepsy patients did not received AED therapy in the first year upon epilepsy diagnosis [32]. In our calendar year-based observation, the percentage of adult prevalent FE patients who did not receive any AED therapy ranged between 8.1% in 2007 to 15.4% in 2014; and it steadily increased over time. The generally lower percentage of the no-AED therapy group in comparison to the study by Ertl et al. [32] can potentially be explained by our conservative sample definition which required at least one AED prescription in the whole observation period. Without knowledge about disease specifics of these patients, the pattern of the steadily increasing percentage of patients with no AED therapy may be explained by several factors: Firstly, epilepsy surgery or treatment with vagus nerve stimulation (VNS) are therapy options for drug-resistant epilepsy patients [33], [34]. Patients with a successful surgery may not need any subsequent AED therapy. Secondly, AED withdrawal may be an option for patients who have been seizure free for several years [33], [35]. Thirdly, non-persistence in AED treatment may also explain why a certain percentage of patients did not receive any AED therapy in the observed follow-up years [36], [37]. Fourthly, it may be possible that an unknown number of patients were diagnosed with FE, received an initial AED therapy, but proved not to need medication, potentially due to an incorrect coding. 

Based on the DDD, a low drug supply was prescribed for all observed drugs. As an example, FE-prevalent monotherapy patients received 171–222 DDDs of supply on average in one observed year only. This may be due to non-adherence of patients to their regime [37]. Another explanation for the low prescribed AED supply may be dosage titration of AED therapy. For example, the DDD for one of the most frequent prescribed AEDs in 2014, LEV, is 1,500 mg per day [28]. However, based on the German label, the lower recommended maintenance dose for adults is 1,000 mg a day, i.e. 0.66 DDDs [25]. If patients achieved seizure freedom with a low AED dosage, which could be the case in a substantial percentage of patients, there is no a need to increase the dose. So, it can be hypothesized that the main explanations for the observed low prescribed AED supply is non-adherence of patients as well as prescription of low AED dosages. Indeed, a significant increase in prescribed dosage in incident FE patients for all types of AED therapy until the third follow-up year was observed. The prescribed dosage remained stable in the fourth follow-up year.

To our knowledge, this is the first study that analyzes the prescription of novel AEDs across all available AEDs in real-world setting in Germany. The data suggest that even if most of these drugs have been available for several years by the time of the observation, a small proportion (<6%) of all adult FE patients received a novel AED therapy. In line with both the label of those drugs and the recommendations of the German Society for Epileptology (Deutsche Gesellschaft für Epileptologie, DGfE), patients received novel AEDs in addition to other AEDs [23], [24], [26], [33], [38]. Furthermore, they received at least 2–3 other AEDs before a novel AED therapy was started. A novel AED is used after several years of disease history and is started in the overwhelming majority of adult FE patients earliest as third-line therapy, in many patients even as fourth- or fifth-line therapy. This study may in fact underestimate this situation as the observation started in 2007, but some of the prevalent FE patients may have received other AEDs before 2007. Thus, this study confirmed that German treatment guidelines are being strongly adhered to, with high prescription of established AEDs, and low prescription of novel AEDs [32]. 

Between the novel AEDs examined in this study (ESL, LCM, PER, and RTG) there were substantial differences in both the number of previously prescribed AEDs and the time from first FE diagnosis until date of first prescription. LCM and ESL are prescribed earlier after first diagnosis, and with fewer previous treatments, compared with PER and RTG. This could be related to the longer experience of the physicians (including knowledge of the safety profile) with ESL and LCM, due to earlier market introduction, compared with PER and RTG. It is also likely that AMNOG assessments of PER and RTG have impacted their use.

### Limitations

Some limitations of this study are acknowledged below. Firstly, patients were included in the analysis only if they received, in addition to a respective FE diagnosis, at least one AED prescription throughout the whole follow-up period. This methodology approach may lead to an underestimation of under-treated FE patients. However, there is some uncertainty around the documented epilepsy diagnoses in our database, especially because the majority of diagnoses were documented in an outpatient setting. Because the principal aim of the analysis was the description of the AED treatment of prevalent FE patients and not the assessment of potential under-treatment, conservative inclusion criteria were chosen to ensure that only patients with a confirmed diagnosis of FE were included. Defining an AED prescription as an inclusion criteria increased the reliability of results as there is a lower probability that patients receiving a FE diagnosis and an AED prescription do not suffer from epilepsy. Nevertheless, epilepsy misdiagnoses cannot be completely excluded, especially as a growing percentage of prevalent FE patients were found to receive no AED therapy in later years. Additionally, because the definition of FE incidence was based on a 6-months washout period, it cannot be completely ruled out that some of the incident FE patients already received their first FE diagnosis or first AED prescription before that washout period.

Secondly, the definition of monotherapy, and of more than one AED therapy was based on a yearly observation of treatment patterns for both prevalent and incident FE patients instead of drug-specific exposure periods. It was assumed that monotherapy was present if only one AED was prescribed throughout the observed respective year. If patients received a monotherapy based on one agent, but switched to a monotherapy with a follow-up AED within one specific year, it was interpreted as therapy with two AEDs. Although nearly 70% of the patients were observed to receive monotherapy, these can be considered the minimum numbers, as the methodology may underestimate those patients switching monotherapies. This also applies for the percentage of patients receiving a therapy with two AEDs versus three AEDs, etc. 

## Conclusions

The study confirms that, in a real-world clinical setting in Germany, most adult patients with FE receive an AED as monotherapy. Novel AEDs are prescribed in less than 6% of the patients and relatively late in the treatment course. These results confirm the recommendations for adult patients from the German Society for Epileptology (Deutsche Gesellschaft für Epileptologie, DGfE) who suggest monotherapy options that do not include the novel AEDs examined in this study.

## Notes

### Abbreviations 

AED: anti-epileptic drug; AMNOG: Arzneimittelmarkt-Neuordnungsgesetz (Act on the Reform of the Market for Medicinal Products); DDD: daily defined dose; FE: focal epilepsy; ICD: International Statistical Classification of Diseases; SD: standard deviation.

### Competing interests

This work was financially supported by UCB Pharma GmbH.

### Acknowledgements

We thank Andreas Fuchs (AOK PLUS, Germany) for administrative support regarding the database and Ulf Maywald (AOK PLUS, Germany) and Hermann-Josef Haeck (UCB Pharma GmbH) for their scientific input and editorial support.

## Figures and Tables

**Table 1:**
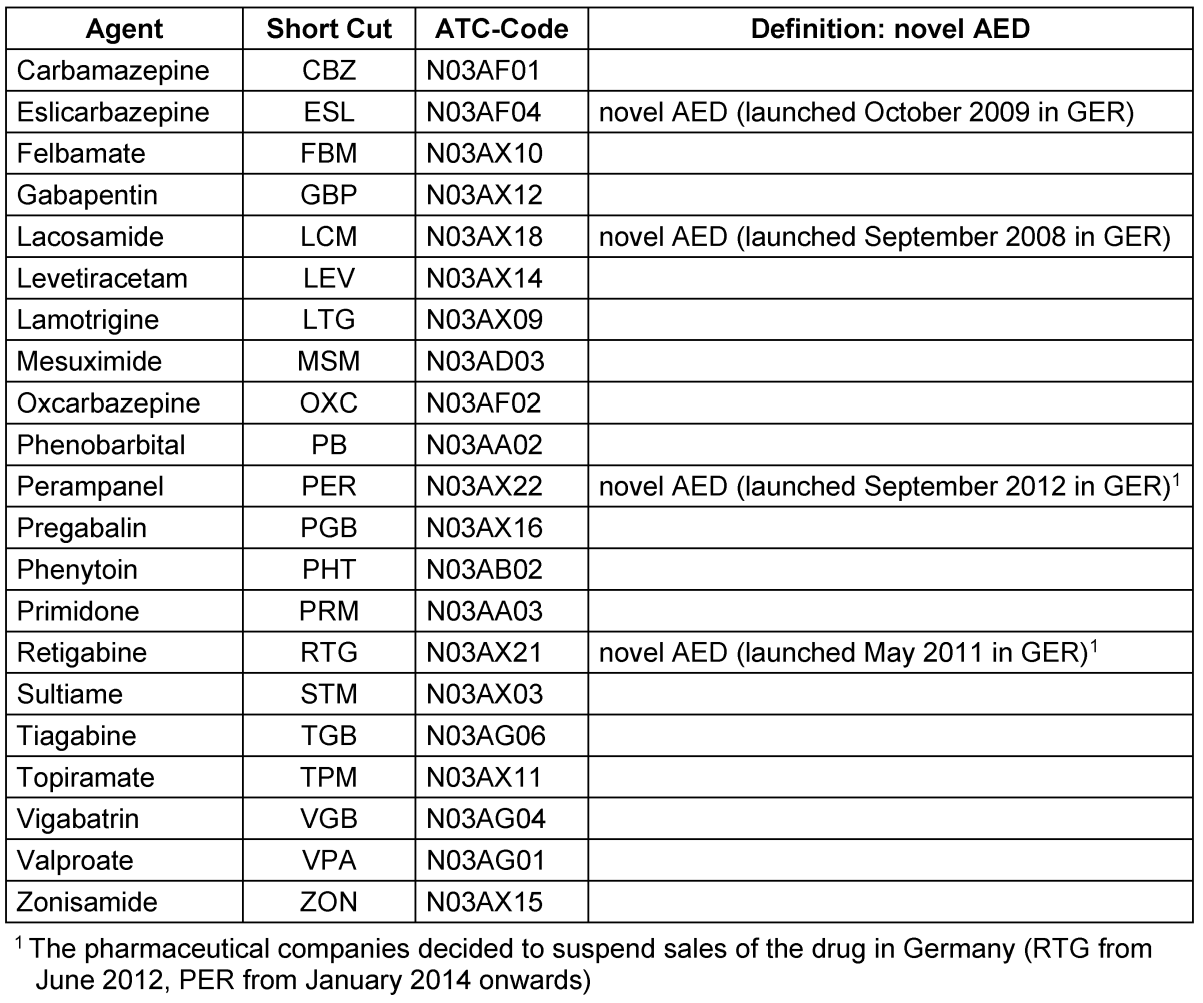
List of all observed anti-epileptic drugs (AEDs)

**Table 2:**
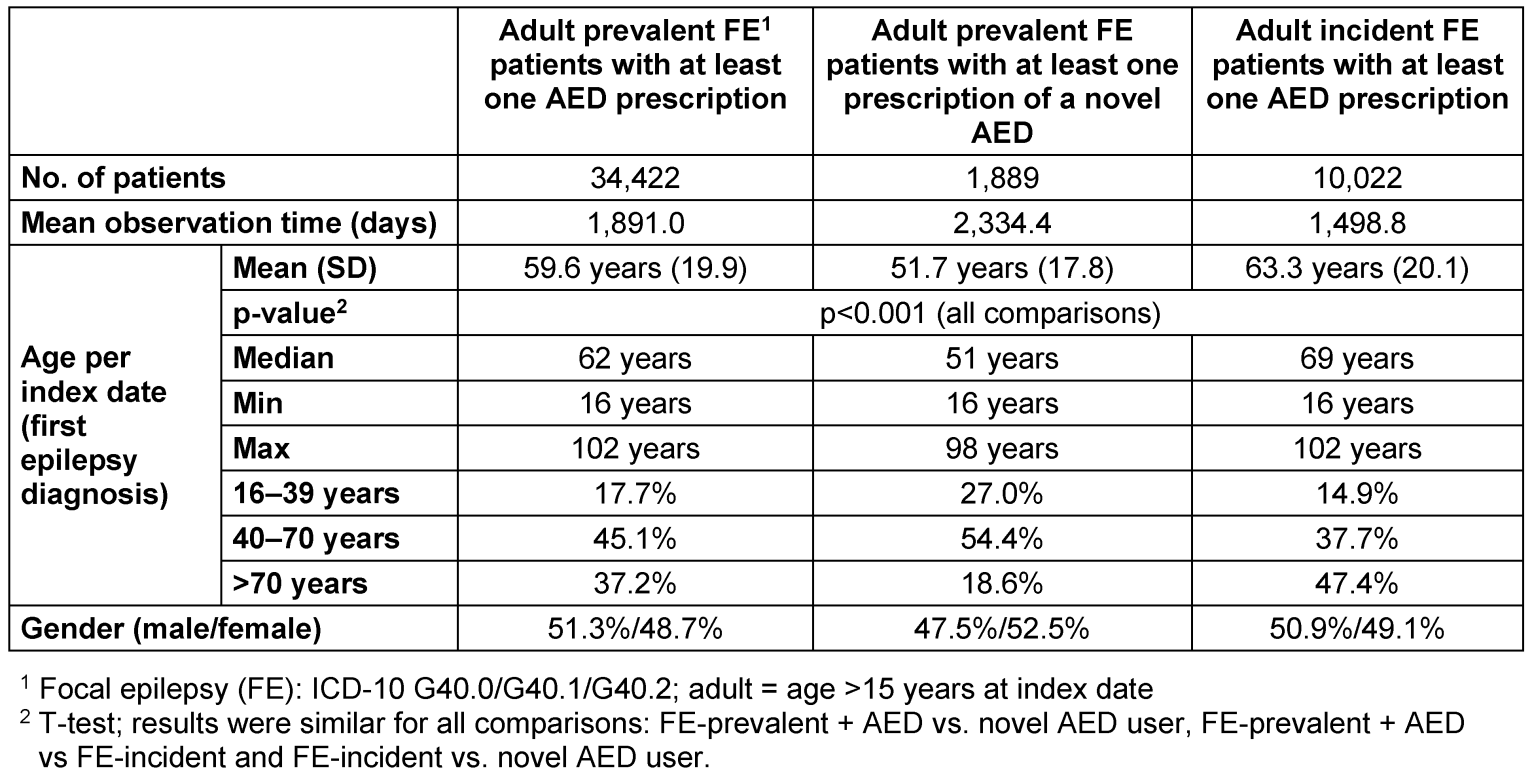
Descriptive characteristics of observed patient samples

**Table 3:**
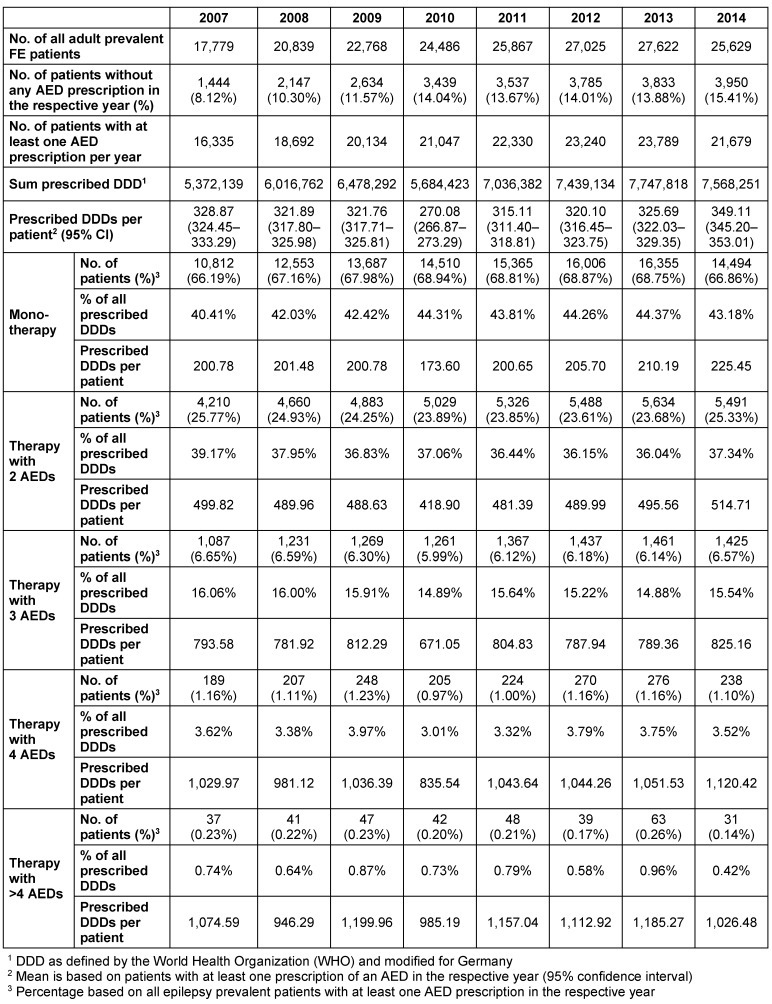
Drug treatment of adult prevalent FE patients

**Table 4:**
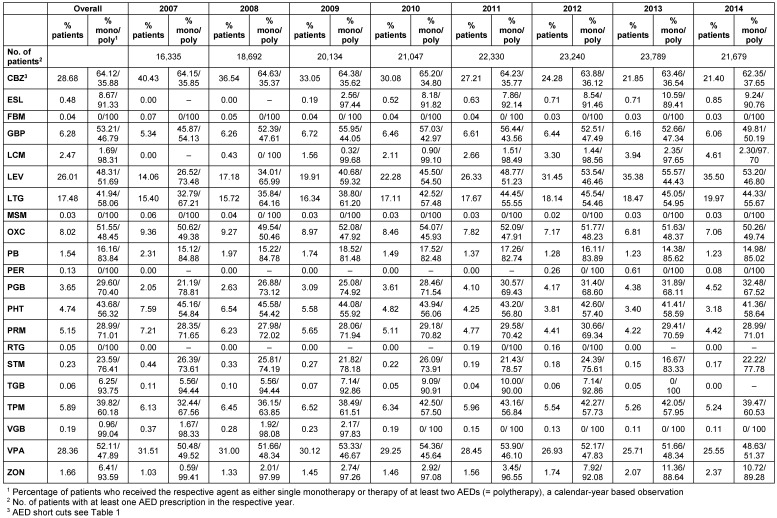
Treatment of adult prevalent FE patients – all prescribed AEDs

**Table 5:**
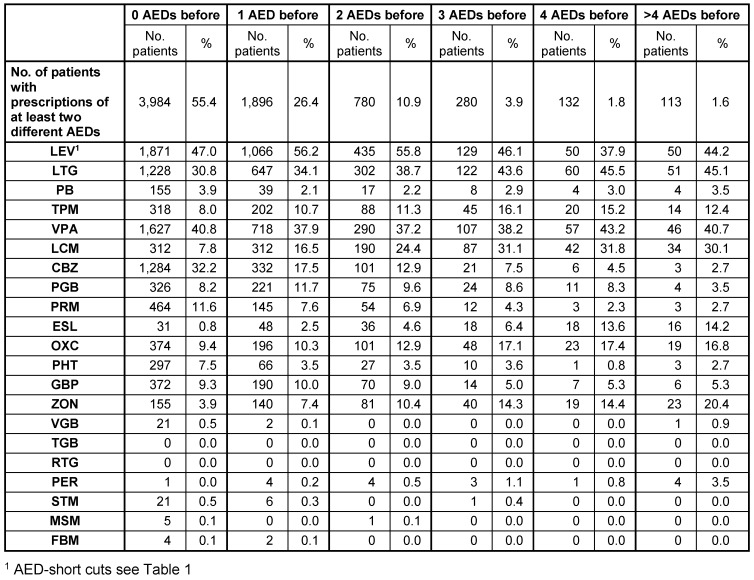
Drug treatment of adult prevalent FE patients with combination therapy in 2014 (at least two different AEDs prescribed in 2014; N=7,185 patients): deviation in number of discontinued AEDs and current treatment

**Table 6:**
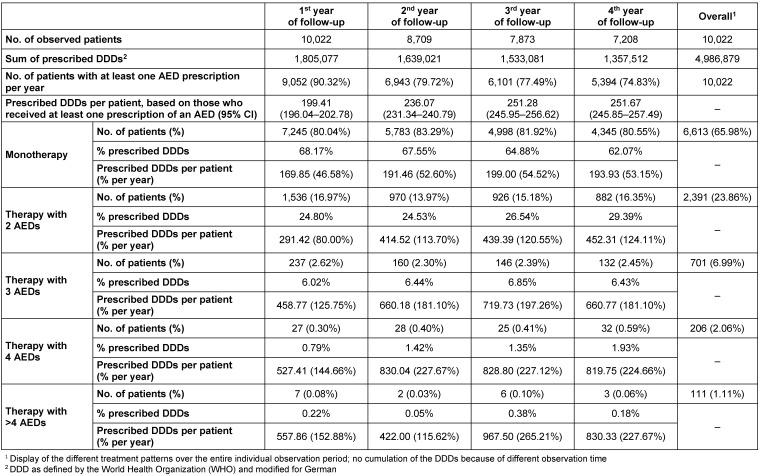
Drug treatment of adult incident FE patients

**Table 7:**
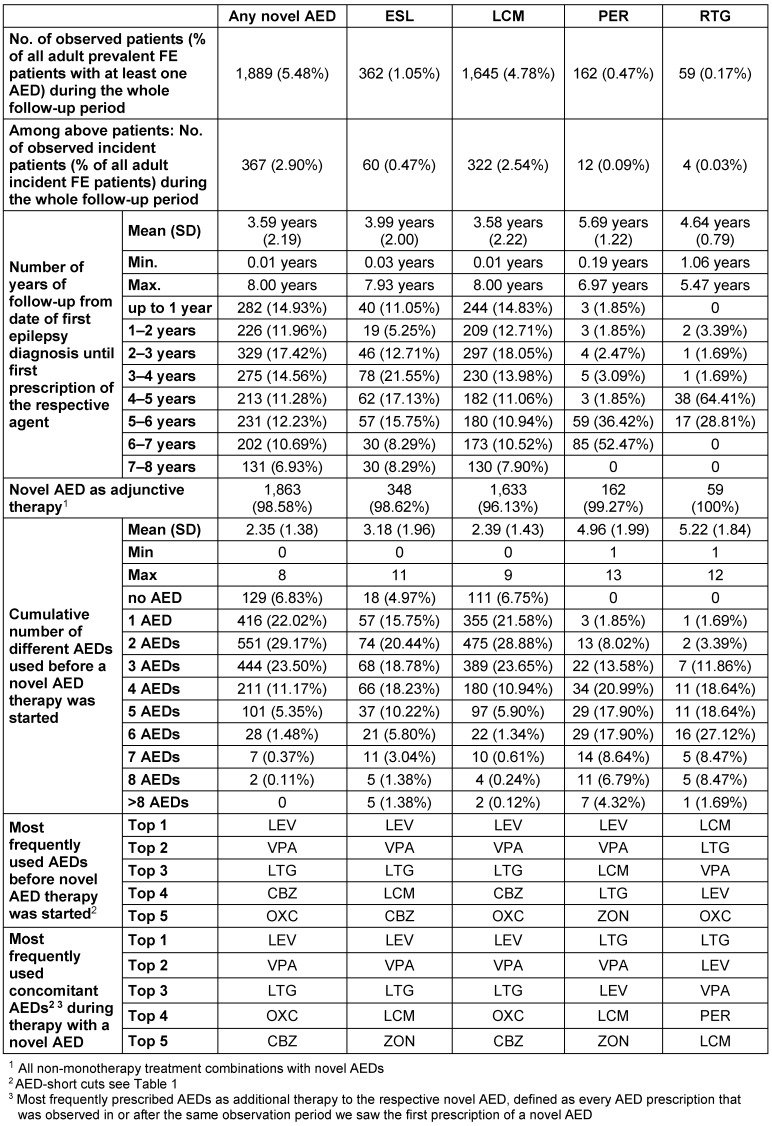
Treatment of adult prevalent FE patients with novel AEDs
